# *Inonotus obliquus* polysaccharide ameliorates serum profiling in STZ-induced diabetic mice model

**DOI:** 10.1186/s13065-021-00789-4

**Published:** 2021-12-17

**Authors:** Tanye Xu, Guodao Li, Xiaobo Wang, Chongning Lv, Yuanyong Tian

**Affiliations:** 1 College of Food Science and Engineering, Dalian Ocean University, Dalian, 116023 Liaoning China; 2 Pharmacy Department, The 967Th Hospital of PLA Joint Logistics Support Force, No. 80 Shengli Road, Xigang District, Dalian, 116021 Liaoning China; 3 School of Traditional Chinese Materia Medica, Shenyang Pharmaceutical University, Shenyang, 110016 Liaoning China

**Keywords:** *Inonotus obliquus* polysaccharide, Diabetes, Hypoglycemic effect, Molecular mechanism, Metabolomics

## Abstract

**Background:**

Diabetes mellitus is a systemic disease mainly caused by the disorder of metabolism, which has become huge threat to human health. Polysaccharides are the main active substance from *Inonotus obliquus (I. obliquus)* with hypoglycemic effect. This study aims to evaluate the hypoglycemic activity and investigate the molecular mechanism of *I. obliquus* polysaccharide (IOP) in streptozotocin (STZ)-induced diabetic mice using metabolomics based on UPLC-Q-Exactive-MS method.

**Results:**

The results showed that the oral administration of IOP in high dose (1.2 g/kg) can significantly reduce the blood glucose with 31% reduction comparing with the diabetic model and relieve dyslipidemia in diabetic mice. By UPLC-Q-Exactive-MS method and multivariate statistical analysis, a total of 15 differential metabolites were identified, including 4 up-regulated and 11 down-regulated biomarkers, of which l-tryptophan, l-leucine, uric acid, 12-HETE, arachidonic acid, PC(20:1(11Z)/14:1(9Z)) and SM(d18:0/24:1(15Z)) were exhibited an important variation, as the potential biomarkers in diabetes. Pathway analysis indicated that phenylalanine, tyrosine and tryptophan biosynthesis and arachidonic acid metabolism were prone to interference in diabetes. Moreover, leucine and proline were reversed and phytosphingosine was further reduced in diabetic mice under the intervention of IOP.

**Conclusion:**

IOP has predominant hyperglycemic effect on STZ-induced diabetic mice via ameliorating serum profiling.

**Supplementary Information:**

The online version contains supplementary material available at 10.1186/s13065-021-00789-4.

## Introduction

Diabetes mellitus is rapidly becoming a global epidemic disease and brings huge threat to human health and social economy. It is estimated that the diabetic patients worldwide are up to 463 million at present [1]. Diabetes mellitus is a systemic disease mainly caused by the disorder of metabolism, which divided into type I and II. Type I diabetes occurs due to the deficiency of insulin secretion caused by the damage of pancreatic *β* cells or other reasons, and type II diabetes is mainly characterized by insulin resistance [2–4]. Hyperglycemia is the most typical symptom of diabetes, moreover, diabetes patients prone to various complication, including hyperlipidemia, hypertension, retinopathy, neuropathy, and nephropathy [5, 6]. Currently chemically synthesized drugs for diabetes mellitus have numbers of serious adverse effects, such as gastrointestinal reaction, hepatic and renal impairment, and high rates of secondary failure [7, 8], and aren’t conducive to the complications treatment. Therefore, development new hypoglycemic agents with minimal adverse effect and excellent efficacy from natural products is still a challenge to the medical system.

*Inonotus obliquus*, also known as “Chaga”, is black-brown plant parasitic fungus and mainly distributed in Europe, Asia, and North America [9–11]. The *I. obliquus* was traditionally used as a folk medicine to treat gastrointestinal cancer, cardiovascular disease and diabetes in Russia, China, and Korea for many years [12–14]. It contains a variety of bioactive substances, including polysaccharides, triterpenes, phenolic acids and flavonoids [15–17]. To date, it has been shown that polysaccharide from *I. obliquus* possessed clear hypoglycemic activity [18, 19]. Several authors have reported that *I. obliquus* polysaccharide (IOP) may exert its antioxidant effect in treating hyperglycemia [20–22], of which the dry matter of culture broth of *I. obliquus* in 500 and 1000 mg/kg body weight showed a significant decrease in blood glucose level in alloxan-induced diabetic mice. Moreover, Wang et al. [23] showed that the effects of IOP on restoration of insulin resistance in streptozotocin-induced type II diabetic mice might involve in the PI3K/Akt signal pathway, which showed significant effect in the dose of 900 mg/kg. Joo et al. [24] suggested that IOP activated adipogenesis of 3T3-L1 preadipocytes and increased PPARγ transcriptional activities, which PPARγ has proved as a key receptor involved in insulin resistance [25]. Despite the remarkably effective of IOP in anti-diabetes, its exact molecular mechanism of the action has not been elucidated. In addition, diabetes mellitus, as a typical metabolic disease, often involves complex metabolic changes, which leads to certain difficulties in the study of pathophysiological process and drug treatment.

Recently, metabolomics has been widely used in pharmacological action and molecule mechanism due to its high sensitivity and comprehensive characterization [26, 27]. Metabolomics focus on the variation of small molecules metabolites in organism, and these differential metabolites may serve as candidate biomarkers of pharmacological efficacy or toxicity [28, 29]. Metabolomics can visually characterize the complex metabolic changes in the development of diabetes and the multi-target actions of traditional Chinese medicine, which has the characteristics of integrity, non-targeting and dynamics [30]. Here, the Q Exactive hybrid quadrupole orbitrap (Q-Exactive) high-resolution mass spectrometry in full scan mode offers a global view of sample extracts, as virtually all ionized compounds are detected with high sensitivity [31].

The purpose of this study was to investigate the anti-diabetic effects of IOP in STZ-induced diabetic mice and the potential mechanism revealed by untargeted metabolomics based on ultra high performance liquid chromatography with Q Exactive hybrid quadrupole orbitrap mass spectrometer (UPLC-Q-Exactive-MS).

## Material and methods

### Materials and reagents

The sclerotia of *I. obliquus* was purchased from Hometown Products Co., Ltd (Jilin, PR China). Streptozocin (STZ) was purchased from Sigma (Saint Louis, Missouri, USA). Metformin was purchased from Sino-American Shanghai Squibb Pharmaceuticals Ltd (Shanghai, China). Blood glucose detection kit was acquired from Solarbio Science & Technology Co., Ltd (Beijing, China). ELISA kits for the determination of total cholesterol (TC), triacylglycerol (TG), low density lipoprotein-cholesterol (LDL-C), high density lipoproteincholesterol (HDL-C) were acquired from Jiancheng Bioengineering Institute (Nanjing, China). Chromatographic grade acetonitrile, methanol, ammonium formate, and formic acid were obtained from Merck (Darmstadt, Germany). Analytical grade ethanol was purchased from the China National Pharmaceutical Group Corporation (Shanghai, China). Ultrapure water (18.2 MΩ) was prepared using a Milli-Q water purification system (Millipore, Shanghai, China).

### The preparation of IOP

The extraction of IOP was followed the method reported previous with the yield of 3.8% [32]. Briefly, the dried powder of *I. obliquus* was conduct with 90% (v/v) ethanol for 20 min at 80 °C twice by ultrasonic technology to remove the lipid soluble substances and pigment components. Then, the dried residue was extracted three times with distilled water for 30 min each at 50 °C via ultrasonic–assisted extraction. After remove the residue, the supernatant was concentrated via vacuum rotary evaporation and then the concentrated solution was precipitated by adding ethanol to final concentration of 75%. After centrifuged at 5000×*g* for 15 min, the IOP was obtained and then freeze-drying.

### Animals and drug administration procedure

Male Kunming mice (6 weeks, 18 g ± 2 g) were obtained from Beijing Experimental Animal Center (Beijing, China) and kept in standard conditions with a normal diet under temperature of 25 ℃ ± 1 ℃ and relative humidity of 55% ± 10% for 1-week adaptive feeding, with 12:12-h light/dark cycle.

Ten healthy mice were selected as normal control group. STZ intraperitoneal injection was used to build the model of diabetes reported by Rajesh et al. [33]. Briefly, mice were injected intraperitoneally with a freshly prepared STZ solution (80 mg/kg body weight) in a citrate buffer (0.1 M, pH4.5). The development of diabetes was confirmed after a week of STZ injection, mice with blood glucose more than 11.1 mmol/L were selected as diabetic mice for further experiment. The diabetic mice were randomly divided into 5 groups of 10 mice each. Metformin group were diabetic mice treated with metformin 0.25 g/kg per day; IOP groups were set into high-, medium- and low-dose (IOPH, IOPM and IOPL groups) as follow: diabetic mice administered with 1.2, 0.8 and 0.4 g/kg per day, respectively. Diabetic model group and normal control group were administered with saline in a matched volume. The experiment was conducted continuously oral administration for 4 weeks and normal diet was given. Animals were anesthetized before eyeball enucleation with intraperitoneal injection of ketamine (80 mg/kg) and xylazine (15 mg/kg). All the serum samples were collected from the orbital sinus for biochemical analysis. Besides, the serum from control, model and IOPH groups were used for metabolomics analysis.

### Oral glucose tolerance test

Oral glucose tolerance test (OGTT) was performed on the last week during experimental period. Mice were fasted for 12 h and serum samples were collected from the tip of the tail vein to measure the fasting blood glucose. Then, all the animals were received glucose (2 g/kg) orally and the blood glucose levels at 30, 60, 90 and 120 min were measured. AUC curve was calculated using GraphPad Prism 5.0 software.

### Biochemical analysis

After oral administration for 4 weeks, blood was collected from the orbital sinus. After centrifugation at 5000×*g* for 10 min at 4 ℃, serum samples were obtained for subsequent analysis. The glucose, TC, TG, LDL-C and HDL-C levels in serum of mice were measured using reagent kits.

### Metabolomics analysis

The serum samples were stored at − 80 ℃ until sample preparation. After thawing the samples on ice, 50 μL serum of each sample was spiked with 450 µL methanol solution. After vertexing for 60 s, the mixture was centrifuged at 10,000×*g* at 4 ℃ for 10 min. The supernatant was injected into the UPLC-Q-Exactive-MS system for metabolomics analysis. The pooled quality control (QC) samples including each serum sample were used to monitor the data acquisition performance during analysis.

An ultra high performance liquid chromatography (UPLC) system (Thermo Fisher Scientific, San Jose, CA, USA) was used to separate the metabolites in the serum. Five microliter aliquots of samples were loaded on a Waters 2.1 mm × 50 mm × 1.7 µm BEH C18 column with a flow rate of 0.25 mL/min. The mobile phase consisted of water containing 0.1% v/v formic acid with 2 mM ammonium formate (A) and acetonitrile (B). Column was maintained at 25 ℃ and eluted with a linear gradient as follows: 5% B at 0–1 min, 5–60% B at 1–5 min, 60–100% B at 5–8 min, 100% B at 8–11 min, 100–60% B at 11–14 min, 60–5% B at 14–15 min, 5% B at 15–18 min. A Q-Exactive mass spectrometer (Thermo Scientific, San Jose, USA) is tandem to the UPLC system equipped with an electrospray ionization (ESI) ion source. Both positive and negative ion modes were applied using full MS scan range 70–1050 m/z with a resolution of 70,000. In MS^2^ mode, the resolution was 35,000, samples were analyzed at 20, 30, 40 NCE (normalized collisional energy). The following parameters were as follows: Spray voltage, 3.5 kV (ESI+), 2.8 kV (ESI−); gas temperature, 350 ℃; sheath gas: 35 arbitrary units; auxiliary gas, 10 arbitrary units; capillary temperature, 320 ℃; S-lens RF, 50. Regarding the sequence of analysis, all the samples were randomly loaded in the instrument.

### Data processing

Statistical analysis was performed on SPSS software version 25. All data were expressed as mean ± standard deviation (M ± SD). Independent samples t-test was used to analyze the differences between groups, with *p* values of less than 0.05 considered statistically significant. The LC–MS/MS raw data files were imported into thermo Scientific TraceFinder software (Thermo Scientific, San Jose, USA) for data pretreatment. The resulting data matrix were obtained with associated retention time, accurate mass, and chromatographic peak area. The data from both positive and negative ion modes were integrated and then imported to SIMCA software Version 14.1 (Umetrics, Umea, Sweden) for multivariate statistical analysis. The pathway analysis was applied in KEGG database (www.genome.jp/kegg). The potential biomarkers were chosen based on their contribution to the variation and correlation within the data set from the S-plots with the variable importance in the projection (VIP) value more than 1.0. The significance was then confirmed via Student t-test with *p* values less than 0.05.

## Results and discussion

### Oral glucose tolerance property

**Fig. 1:**
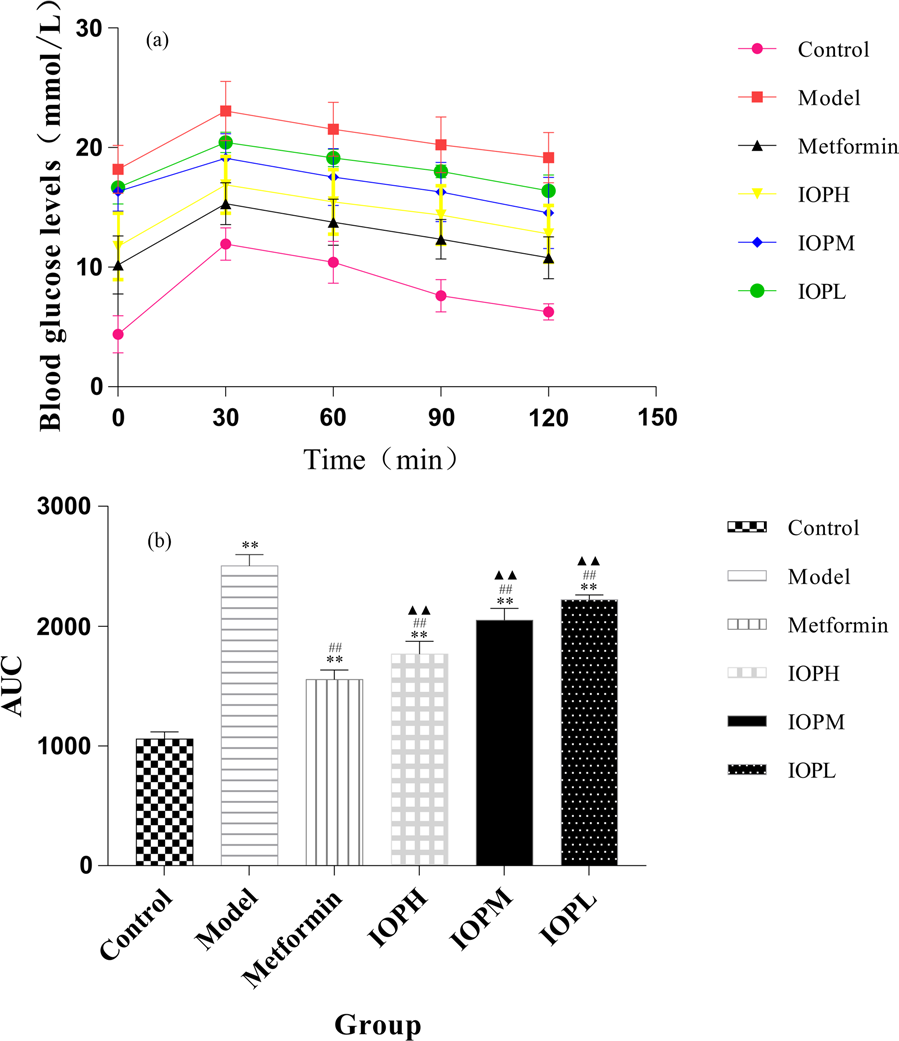
Effects of IOP on oral glucose tolerance test **a** at 0, 30, 60, 90, 120 min and area under curve **b** in the experimental mice

After oral administration of glucose, the blood glucose levels of all the experimental mice were measured to investigate the glucose tolerance ability, which was shown in Fig. 1a, the detailed data are shown in supplementary information files (Additional file 3: Table S1). The blood glucose levels of all mice reached the peaks at 30 min. Compared with the highest level of diabetic model group at 23.05 mmol/L, the blood glucose levels of IOP groups in high-, medium-, and low-dose were much lower (p < 0.05). Then the control group turned gradually to the normal level with 47.4% decreased after oral administration of glucose at 120 min. Meanwhile, IOP administration of mice in high-, medium-, and low-dose exhibited 24.3%, 23.8% and 19.9% reduction respectively, comparing with their peak glucose levels. However, the blood glucose levels of diabetic mice were consistently maintained in a high level throughout the whole time. The area under the curve (AUC) of mice after oral administration of glucose was shown in Fig. 1b. The model group showed the largest AUC among the six groups. The AUCs were significantly decreased in the administration of IOP (IOPH, IOPM and IOPL groups) compared to the model group (p < 0.01).

### Fasting blood glucose, TC, TG, LDL-C and HDL-C levels in serum

In addition to hyperglycemia, dyslipidemia is also a prominent risk factor of the progression of diabetes, mainly in the increased in TCHO, TG and LDL-C levels and decreased in HDL-C level in serum. Table 1 shows the results of fasting blood glucose, TC, TG, LDL-C and HDL-C levels in serum of the mice. In this experiment, the fasting blood glucose levels were exhibited an extremely significant uptrend in the diabetic model group compared with the normal control group (p < 0.01). Further, the treatment of metformin and IOP in high- and medium-dose all showed a significant downtrend of fasting blood glucose levels comparing with the diabetic model group. The high-dose of IOP can decrease 31% blood glucose in diabetic mice. Moreover, the TCHO, TG and LDL-C levels were elevated and HDL-C levels were decreased in the model group, which were consisted with previous study [23]. After the treatment of IOP in high-dose, a remarkable relief of lipid profiles disorder was observed (p < 0.01), which had the same therapeutic effects of metformin.

**Table 1:** The levels of glucose, TCHO, TG, LDL-C and HDL-C in serum of mice

Parameter (mmol/L)	Control	Model	Metformin	IOPH	IOPM	IOPL
Glucose	5.34 ± 0.68	12.51 ± 1.3^**^	7.57 ± 1.31^**##^	8.59 ± 1.55^**##^	10.41 ± 0..9^**#▲▲^	11.39 ± 1.55^**▲▲^
TCHO	2.87 ± 0.1	4.69 ± 0.39^**^	3.24 ± 0.36^##^	3.34 ± 0.39^*##^	4.04 ± 0.25^**#▲▲^	4.3 ± 0.47^**▲▲^
TG	1.68 ± 0.12	3.08 ± 0.22^**^	2.08 ± 0.31^*##^	2.23 ± 0.42^*##^	2.53 ± 0.42^**#^	2.68 ± 0.31^**#▲^
LDL-C	0.47 ± 0.06	2.58 ± 0.28^**^	1.24 ± 0.33^**##^	1.15 ± 0.33^**##^	1.51 ± 0.36^**##^	1.74 ± 0.46^**##^
HDL-C	1.04 ± 0.12	0.35 ± 0.09^**^	0.81 ± 0.12^*##^	0.77 ± 0.22^*##^	0.59 ± 0.21^**^	0.52 ± 0.11^**#▲▲^

VS control group, * means significant difference (*p* < 0.05); ** means extremely significant difference (*p* < 0.01)

VS model group, ^#^ means significant difference; ^##^ means extremely significant difference

VS metformin group, ^▲^ means significant difference; ^▲▲^ means extremely significant difference

et sequentia

### Multivariate statistical analysis and identification of differential metabolites

**Fig. 2:**
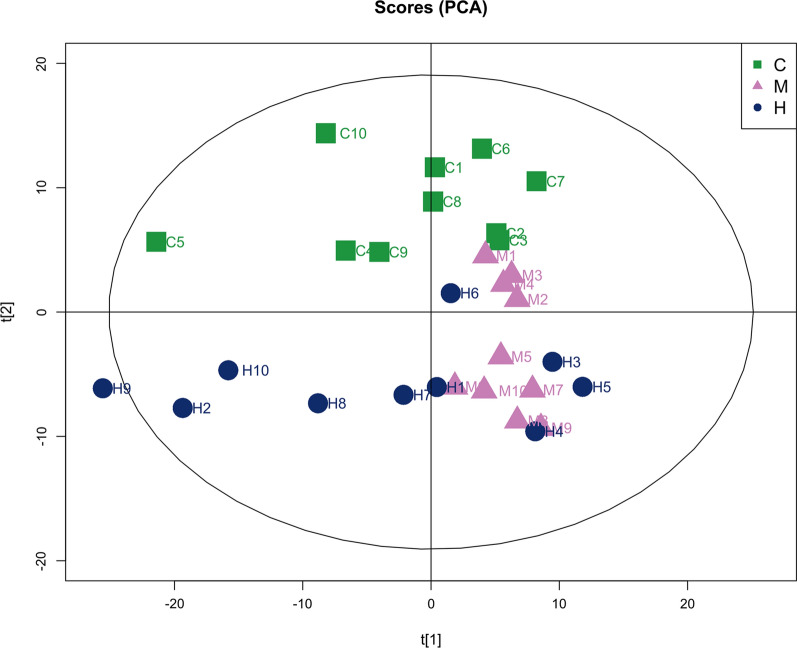
The PCA score plot of serum samples from C (control group), M (model group), and H (IOPH group)

**Fig. 3:**
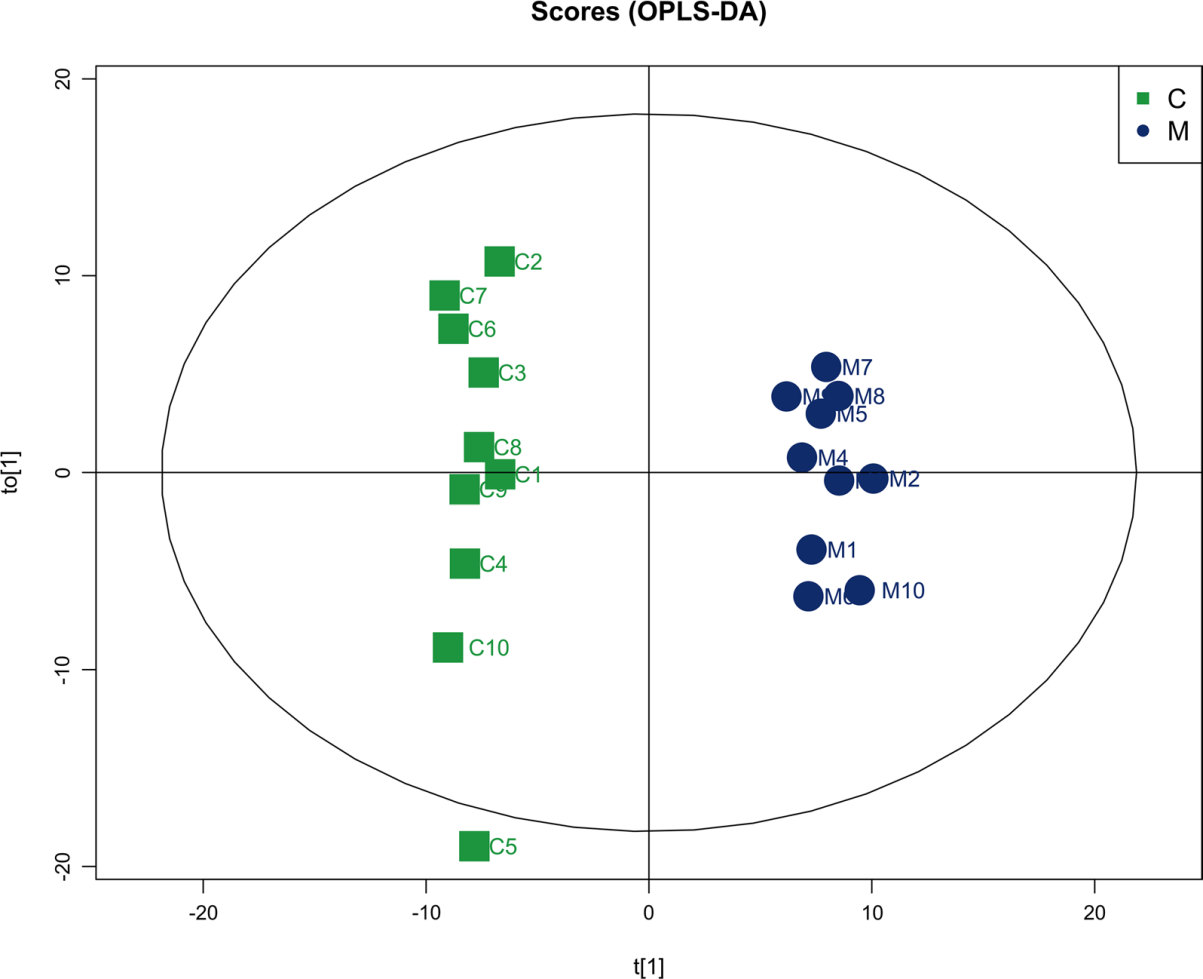
OPLS-DA score plot of serum samples from C (control group) and M (model group)

**Fig. 4:**
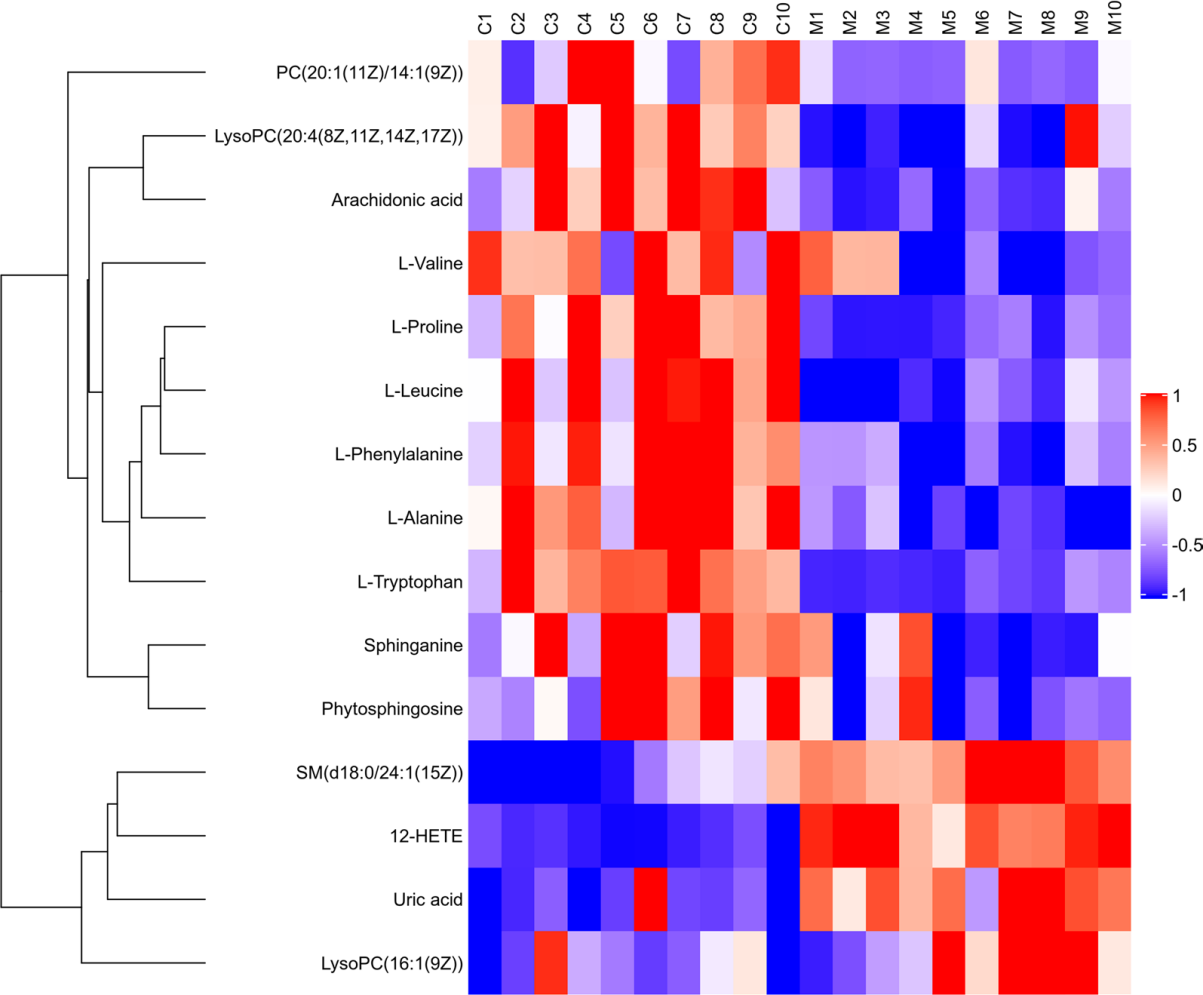
A heat map demonstrating the trend of metabolites variation in C (control group) and M (model group)

**Fig. 5:**
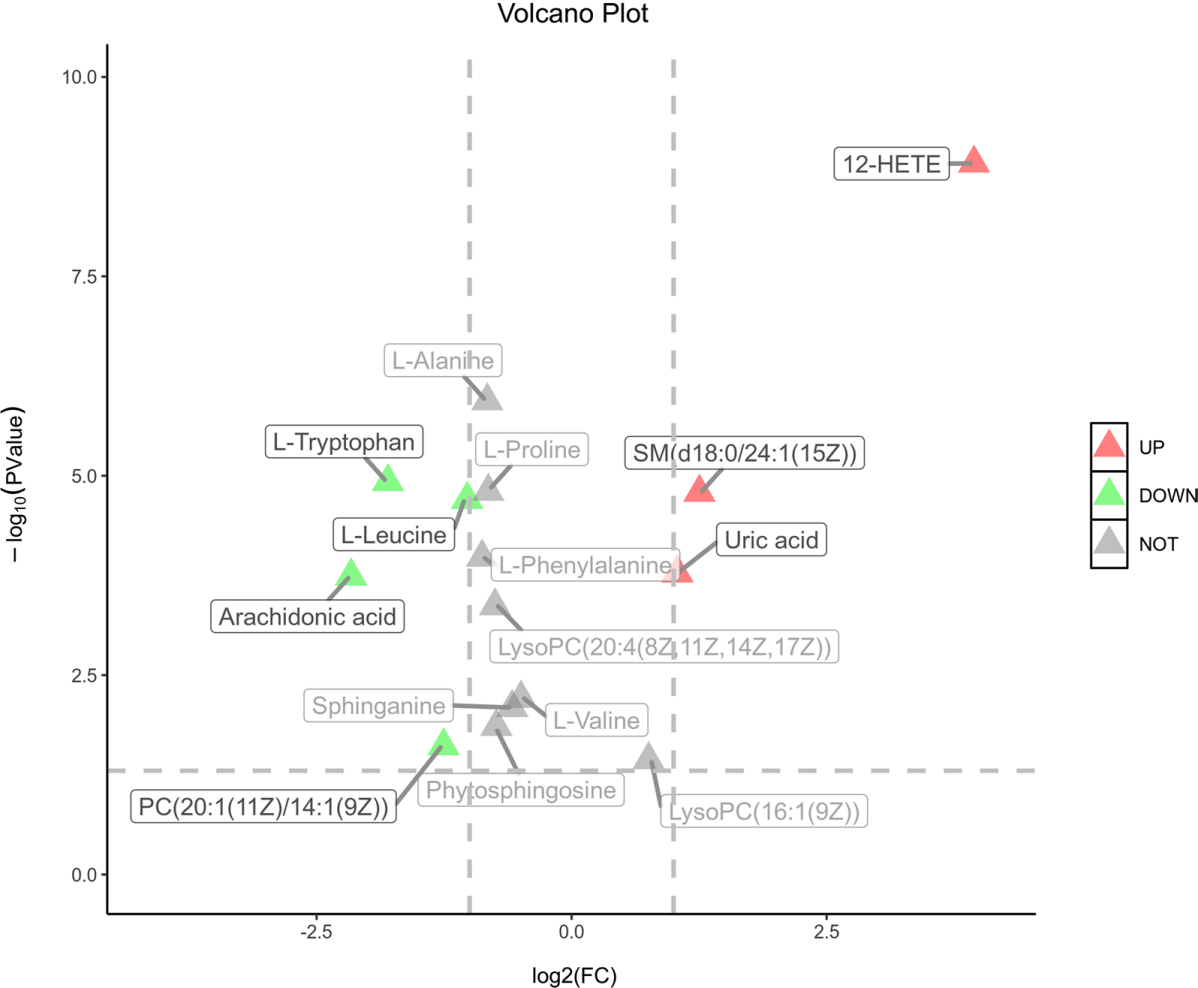
A volcano plot of differential metabolites, seven marked metabolites with more than twofold change (|log2 FC|> 1) and P < 0.01 (− Log10 P > 2)

**Fig. 6:**
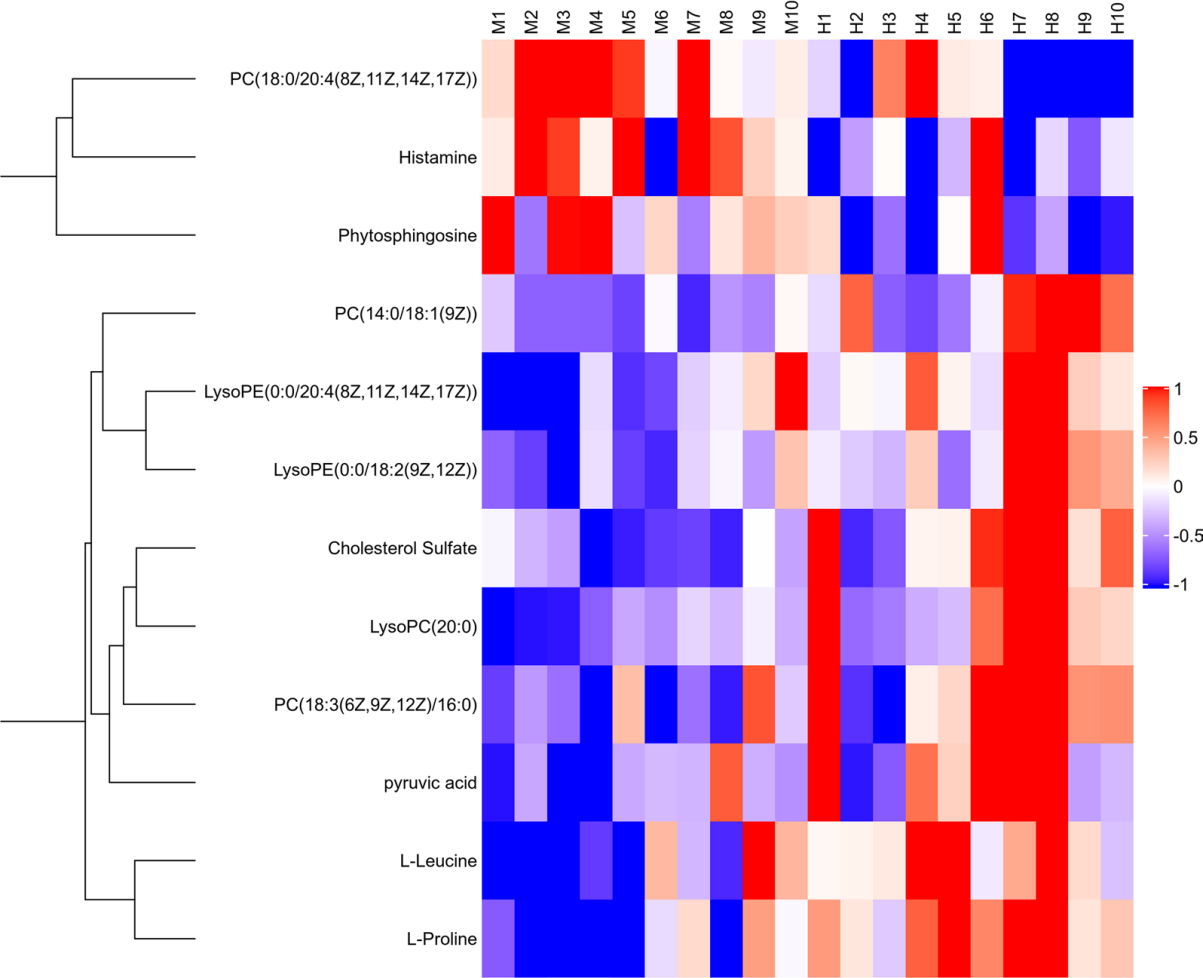
A heat map demonstrating the trend of metabolites variation in M (model group) and H (IOPH group)

**Fig. 7:**
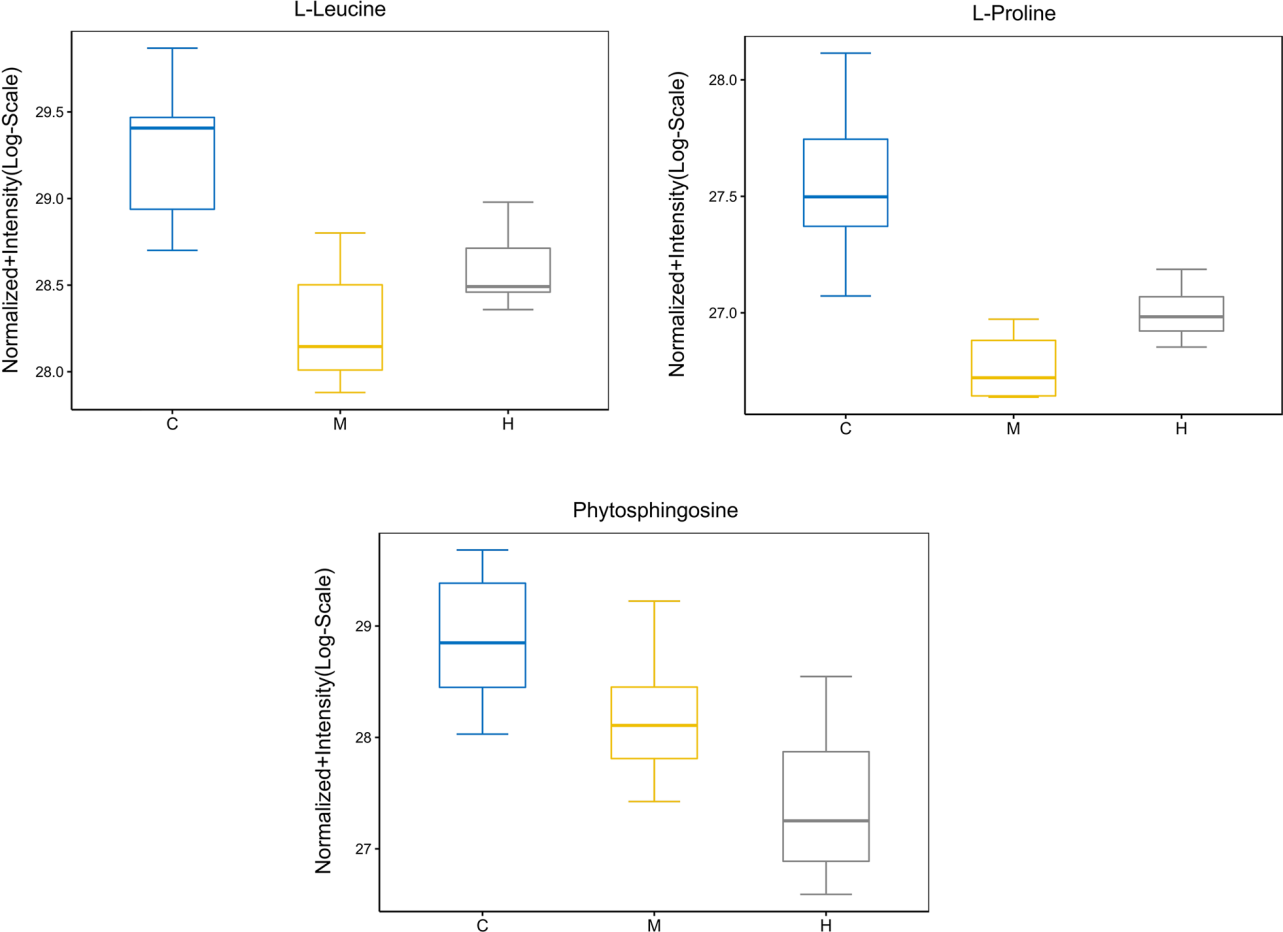
A set of boxcharts in three metabolites which significant changed in diabetic mice and regulated by IOP

**Fig. 8:**
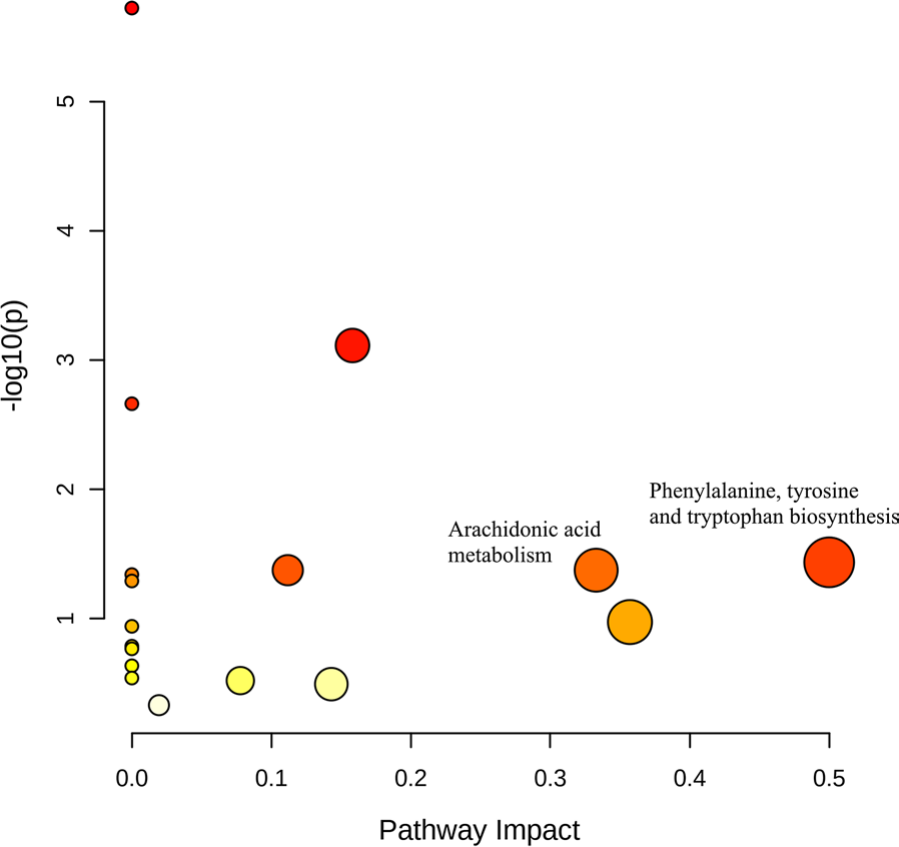
Pathway analysis of metabolite sets between control and model groups

**Fig. 9:**
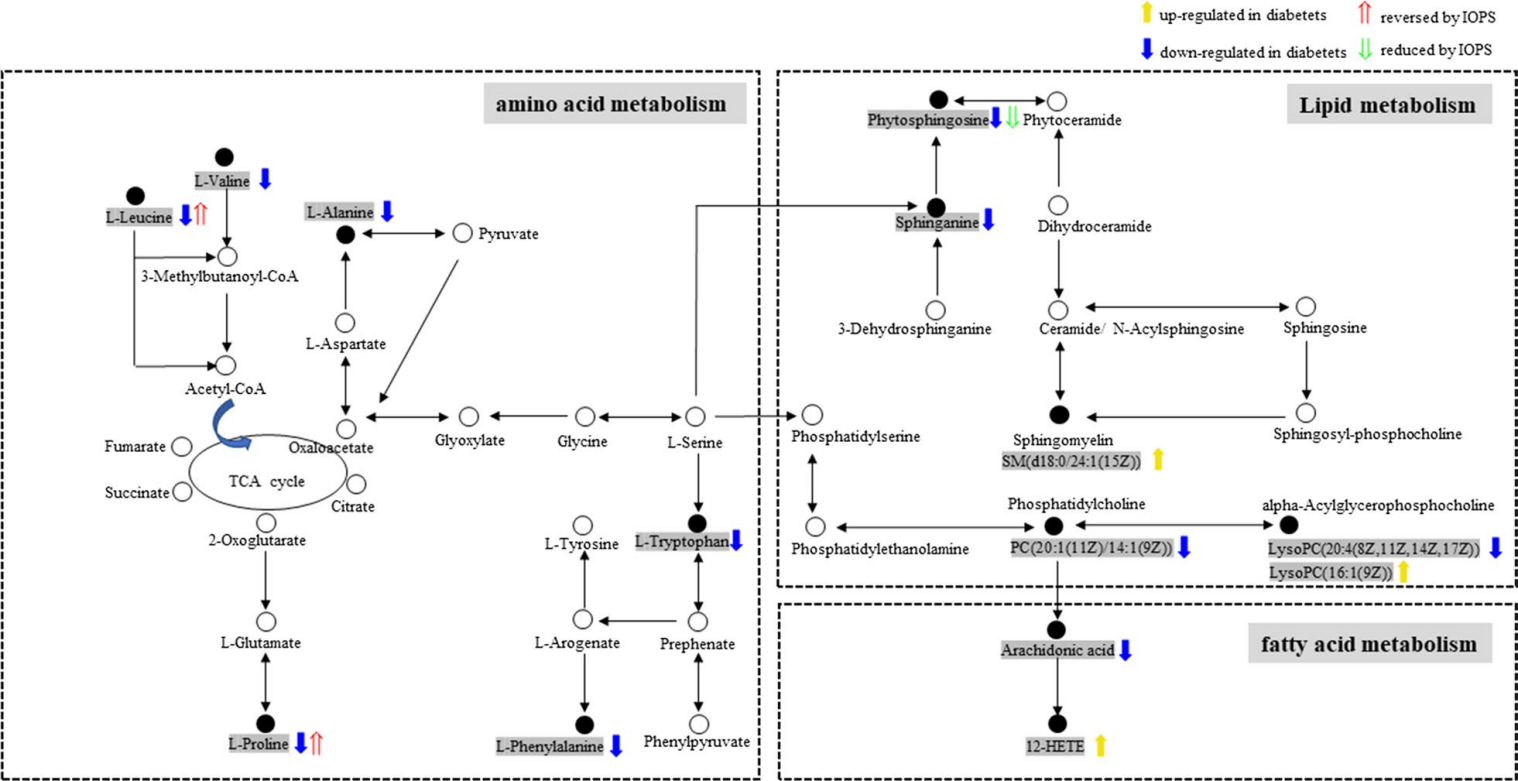
The metabolic pathway network of the pathological processes of diabetes and the intervention of IOP on the molecular levels

All data matrix acquired from the LC–MS analysis platform were pre-processing by TraceFinder Software. Ion peaks presenting in at least 50% of any group were retained for subsequent analysis. Thus, a total of 508 compounds were detected and identified in both positive and negative ion modes in the mass spectrometric data by comparing to the database. The total ion chromatograms (TICs) of the QC samples showed good reproducibility in intensity and retention time of ion peaks in both positive and negative ion mode (shown in Additional file 1: Fig. S1 and S2). Multivariate statistical analyses are widely applied in the metabolomics analysis process, of which Principal Component Analysis (PCA) is an unsupervised pattern recognition method which used to analyze a general overview of the main discriminants among these samples. In the PCA analysis of three groups (shown in Fig. 2), the control group (C group) showed a good distinction with others, while there was a blurred boundary among the areas occupied by the diabetic model group (M group) and IOPH group (H group). Therefore, a supervised pattern recognition method, OPLS-DA was used to discover the differences between M group and C/H group. The OPLS-DA model of control and model groups showed clearly distinguishable (Fig. 3). sevenfold cross-validation was conducted to ensure the robustness of the model with the parameters of R^2^X = 0.252, R^2^Y = 0.981, and Q^2^ = 0.949. Then, 15 significant differential metabolites were screened by comparing the model with control group. Heat maps of these differential metabolites are shown in Fig. 4. Among them, 4 metabolites were upregulated while 11 metabolites were downregulated in the diabetic mice. Moreover, Table 2 shows the detailed results of differential metabolites in the C and M groups. A volcano plot of differential metabolites was obtained via log 2 (fold change of the metabolite) as abscissa and the -log10 (*p* value of the metabolite) as ordinate. As seen from Figs. 5, 7 marked metabolites, l-tryptophan, l-leucine, uric acid, 12-HETE, arachidonic acid, PC(20:1(11Z)/14:1(9Z)) and SM(d18:0/24:1(15Z)) were those exhibited an important variation between C and M groups with more than twofold change (|log2 FC|> 1) and *p* < 0.01 (−Log10 *p* > 2). In the following analysis of OPLS-DA model between M and H group (shown in Additional file 2: Fig. S3), 12 significant differential metabolites were screened and the heat maps was displayed in Fig. 6. Comparing with the above 15 significant differential metabolites selected in diabetic mice, there were three metabolites significantly altered, of which leucine and proline were reversed and phytosphingosine was further reduced in diabetic mice under the intervention of IOP. A set of boxcharts of the three metabolites visualize the contents variations in C, M and H groups, which was shown in Fig. 7. Pathway analysis indicated that phenylalanine, tyrosine and tryptophan biosynthesis and arachidonic acid metabolism were prone to interference in diabetes (shown in Fig. 8). To further explore the pathological processes of diabetes and the intervention of IOP, a network of the metabolism involved in amino acid metabolism, lipid metabolism and fatty acid metabolism was drawn based on the KEGG database (shown in Fig. 9).

**Table 2:** Significant metabolites between C and M group

No.	Rt/min	Ion mode	m/z	Formula	Metabolites	VIP	Fold change (M/C)	Related pathway
1	0.55	[M + H] +	90.05	C_3_H_7_NO_2_	l-Alanine	1.62	0.56^#^	Amino acid metabolism
2	0.55	[M + H]^+^	116.07	C_5_H_9_NO_2_	l-Proline	1.77	0.57 ^#^	Amino acid metabolism
3	0.55	[M + H]^+^	118.09	C_5_H_11_NO_2_	l-Valine	1.93	0.71 ^#^	Amino acid Metabolism
4	0.58	[M-H]^−^	167.02	C_5_H_4_N_4_O_3_	Uric acid	2.07	2.06 ^#^	Purine metabolism
5	0.60	[M + H]^+^	132.10	C_6_H_13_NO_2_	l-Leucine	3.62	0.49 ^#^	Amino acid metabolism
6	0.66	[M + H]^+^	166.09	C_9_H_11_NO_2_	l-Phenylalanine	1.59	0.54 ^#^	Amino acid metabolism
7	2.78	[M + H]^+^	205.10	C_11_H_12_N_2_O_2_	l-Tryptophan	1.89	0.29 ^#^	Amino acid metabolism
8	5.95	[M + H]^+^	318.30	C_18_H_39_NO_3_	Phytosphingosine	2.00	0.60 *	Lipid metabolism
9	6.65	[M + H]^+^	302.31	C_18_H_39_NO_2_	Sphinganine	1.18	0.67 ^#^	Lipid metabolism
10	6.71	[M + H]^+^	494.32	C_24_H_48_NO_7_P	LysoPC(16:1(9Z))	1.68	1.69 *	Lipid metabolism
11	6.90	[M + H]^+^	544.34	C_42_H_80_NO_8_P	LysoPC(20:4(8Z,11Z,14Z,17Z))	3.77	0.59 ^#^	Lipid metabolism
12	7.43	[M-H]^−^	319.23	C_20_H_32_O_3_	12-HETE	3.89	15.36 ^#^	Fatty acid metabolism
13	8.70	[M-H]^−^	303.23	C_20_H_32_O_2_	Arachidonic acid	1.65	0.22 ^#^	Fatty acid metabolism
14	9.32	[M + H]^+^	758.57	C_42_H_80_NO_8_P	PC(20:1(11Z)/14:1(9Z))	8.72	0.42 *	Lipid metabolism
15	13.13	[M + H]^+^	815.70	C_47_H_95_N_2_O_6_P	SM(d18:0/24:1(15Z))	1.79	2.38 ^#^	Lipid metabolism

^*^P < 0.05, ^#^P < 0.01

### Interpretation of differential metabolites

In this experiment, the serum profiling revealed the metabolism disorders in diabetic mice. In the amino acid metabolism, the six amino acids l-leucine, l-valine, l-tryptophan, l-phenylalanine, l-proline and l-alanine were decreased in diabetic mice with the negative correlations with blood glucose, which verified the previous demonstrations [34, 35]. Moreover, it is considered that branched chain amino acid leucine, valine, and isoleucine can improve glucose homeostasis and have the synergism effect on glucose homeostasis [36, 37]. l-Phenylalanine is the precursor of tyrosine and can improve glucose tolerance in rats by reducing plasma ghrelin and stimulating insulin release [38]. Mirsky et al. [39] suggested that l-tryptophan can significantly decrease the blood sugar concentration of normal rats through oral administration. The tryptophan disordered metabolite xanthurenic acid is concerned with the hyperglycemia due to the ability to bind insulin [40, 41]. Researches showed that l-proline had the hypoglycemic effect in experimental diabetic rats by improving the nerve growth factor and brain-derived neurotrophic factor [42]. l-Alanine is an important participant as well as a regulator of glucose metabolism and it was said that the mechanism of hypoglycemic action of biguanides may be a reduction in the Na+/l-alanine transport system [43]. Moreover, l-alanine is important in immune competence. After the administration of IOP, the contents of l-leucine and l-proline were reversed comparing with those in M group. Some evidence indicates that leucine stimulates protein synthesis in pancreatic *β* cell, which as a potentially important leucine-target tissue [44, 45]. Additionally, it was also reported that leucine supplementation could improve glucose tolerance in mice [46, 47]. This may explain the reversion of leucine in H group.

In the lipid metabolism, our data displayed decreased levels of phytosphingosine and sphinganine in diabetic mice, which were consistent with previous results [48]. According to the reports, sphinganine were identified to be significantly associated with diabetes mellitus and cardiovascular complications [49, 50]. As to phytosphingosine, its content was further reduced in the administration of IOP comparing with the model group. Lysophosphatidylcholine (LysoPC), the important intermediate products in the lipid metabolism, is referred to be concerned with the formations of cholesterol and low-density lipoprotein [51] and considered as bioactive medium in the process inflammation and increase oxidative stress [52, 53]. In general, phosphatidylcholine (PC) can transform into LysoPC via lecithin-cholesterol acyltransferase in plasma and participate in the production of arachidonic acid [54]. In this study, the decreased levels of PC(20:1(11Z)/14:1(9Z)) and LysoPC(20:4(8Z,11Z,14Z,17Z)) and the increased levels of SM(d18:0/24:1(15Z)) and LysoPC(16:1(9Z)) were also observed in diabetic mice. The result of up-regulation in LysoPC(16:1(9Z)) was similar to that in previous report [55]. Meanwhile, the lipid metabolism disturbance was proved to be involved in the pathogenesis of diabetic retinopathy [56].

Arachidonic acid is a polyunsaturated, essential fatty acid that mediates inflammation in organism. Arachidonate lipoxygenase enzymes metabolize arachidonic acid to generate potent inflammatory mediators and play an important role in inflammation-associated diseases [57]. Studies showed that arachidonic acid can reverse the decrease of prostaglandin E2 content in the incubation media of diabetic retinas [58]. It is frequently reported that arachidonic acid and its metabolite 12-hydroxyeicosatetraenoic acid (12-HETE) are closely related to the diseases of inflammation and oxidative stress [57, 59]. It was reported that 12-HETE participated in macrophage recruitment in islets and pro-inflammatory cytokine mediated islet damage in type 1 diabetic mouse models [60, 61], showing an inhibition of insulin secretion and metabolic activity [62]. Same to previous researches [63, 64], an increase of 12-HETE and a decrease of arachidonic acid were observed in hyperglycemia in this experiment, which may show the metabolic disorders associated with inflammation.

## Conclusions

In this study, a metabolomics technique based on UPLC-Q-Exactive-MS method was used to illustrate the underlying mechanism of pathophysiological process in diabetes and therapeutic effect of IOP. A total of 15 differential metabolites were identified by comparing the normal control group with diabetic model group, including 4 up-regulated and 11 down-regulated biomarkers, of which l-tryptophan, l-leucine, uric acid, 12-HETE, arachidonic acid, PC(20:1(11Z)/14:1(9Z)) and SM(d18:0/24:1(15Z)) were exhibited an important variation and can be seen as the potential biomarkers in diabetes. Pathway analysis indicated that phenylalanine, tyrosine and tryptophan biosynthesis and arachidonic acid metabolism were prone to interference in diabetes. After oral administration of IOP, the blood glucose was declined and the lipid metabolism disorder was alleviative. Moreover, the differential metabolites leucine and proline were reversed and phytosphingosine was further reduced in diabetic mice under the intervention of IOP in the dose of 1.2 g/kg. The metabolomics approach in this study would lead to a further understanding on the pathophysiological process in diabetes and thus facilitate target screening for therapeutic intervention. Additionally, clarifying the metabolic regulation effect of IOP at molecular level could provide scientific evidence for hypoglycemic effect.

## Supplementary Information

**Additional file 1.** The total ion chromatogram (TIC) of six QC samples in both positive and negative ion modes.

**Additional file 2.** OPLS-DA score plot of serum samples from in M (model group) and H (IOPH group).

**Additional file 3.** The OGTT detailed results of mice.

## Data Availability

All data generated or analysed during this study are included in this published article and its supplementary information files.

## Abbreviations

IOP: *Inonotus obliquus* polysaccharide
UPLC-Q-Exactive-MS: Ultra high performance liquid chromatography with Q Exactive hybrid quadrupole orbitrap mass spectrometer
TC: Total cholesterol
TG: Triacylglycerol
LDL-C: Low density lipoprotein-cholesterol
HDL-C: High density lipoprotein-cholesterol
STZ: Streptozotocin
LysoPC: Lysophosphatidylcholine
PC: Phosphatidylcholine
SM: Sphingomyelin
12-HETE: 12-Hydroxyeicosatetraenoic acid
