# Supplementary material for: Inonotus obliquus polysaccharide ameliorates serum profiling in STZ-induced diabetic mice model
Source: BMC Chem. 2021 Dec 17;15(1):64. doi: 10.1186/s13065-021-00789-4 (PMC8684258; doi:10.1186/s13065-021-00789-4)
Supplement: Supplementary file 1 — Additional file 1. The total ion chromatogram (TIC) of six QC samples in both positive and negative ion modes. [file 13065_2021_789_MOESM1_ESM.docx]

Fig. S1 The total ion chromatogram (TIC) of six QC samples in positive ion mode.

Fig. S2 The total ion chromatogram (TIC) of six QC samples in negative ion mode.
