# Supplementary figures and images for: Inonotus obliquus polysaccharide ameliorates serum profiling in STZ-induced diabetic mice model
Source: BMC Chem. 2021 Dec 17;15(1):64. doi: 10.1186/s13065-021-00789-4 (PMC8684258; doi:10.1186/s13065-021-00789-4)

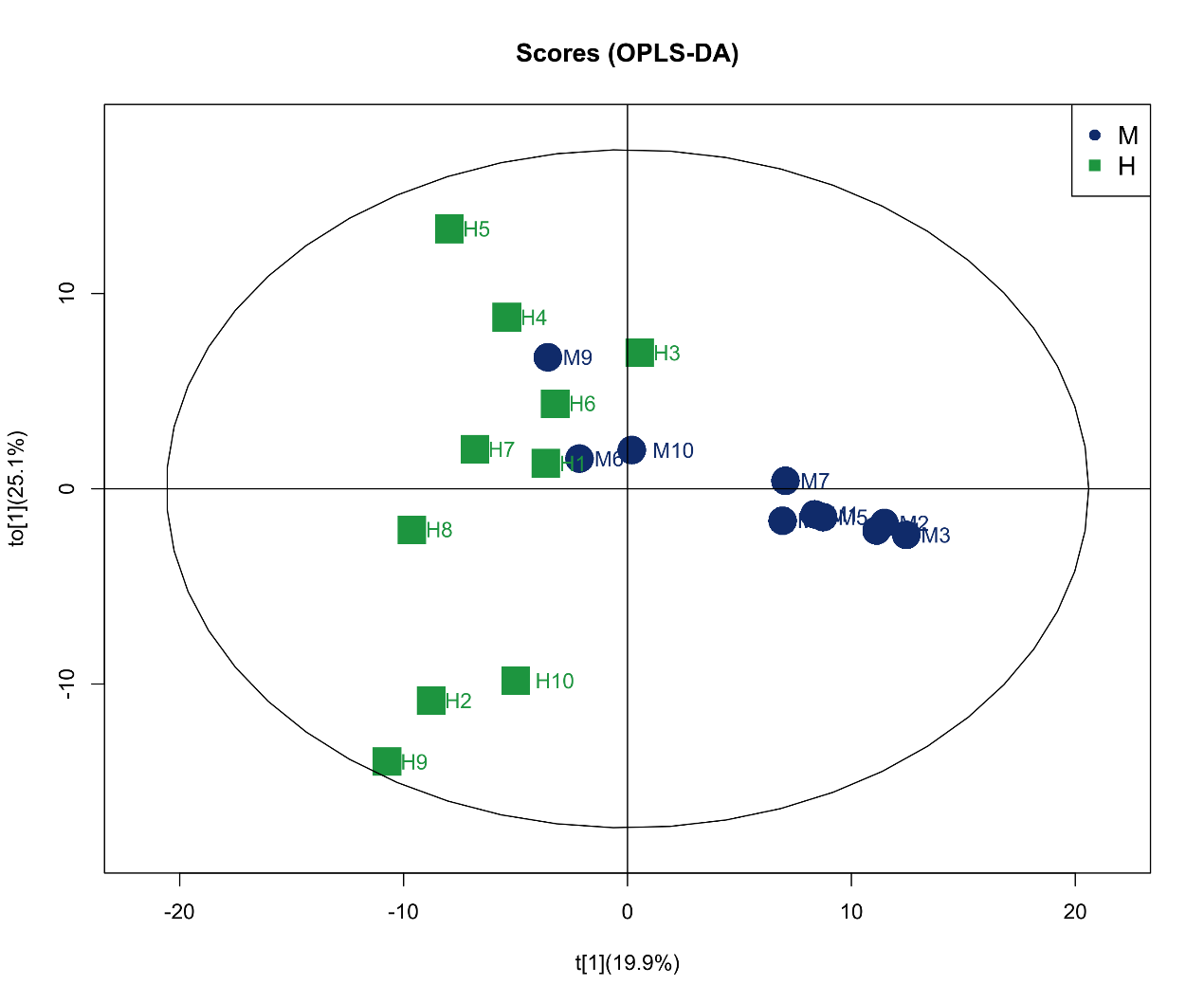


Fig. S3 OPLS-DA score plot of serum samples from in M (model group) and H (IOPH group).

Supplement: Supplementary file 2 — Additional file 2. OPLS-DA score plot of serum samples from in M (model group) and H (IOPH group). [file 13065_2021_789_MOESM2_ESM.docx]
