# Supplementary material for: Inonotus obliquus polysaccharide ameliorates serum profiling in STZ-induced diabetic mice model
Source: BMC Chem. 2021 Dec 17;15(1):64. doi: 10.1186/s13065-021-00789-4 (PMC8684258; doi:10.1186/s13065-021-00789-4)
Supplement: Supplementary file 3 — Additional file 3. The OGTT detailed results of mice. [file 13065_2021_789_MOESM3_ESM.docx]

Table S1 The OGTT detailed results of mice

| Group | 0 min | 30 min | 60 min | 90 min | 120 min |
| --- | --- | --- | --- | --- | --- |
| Control | 4.40±1.43 | 11.93±1.24^※※^ | 10.40±1.61^※※^ | 7.60±1.23^※※^ | 6.27±0.62^※^ |
| Model | 18.20±1.83** | 23.05±2.26**^※※^ | 21.53±2.06**^※^ | 20.23±2.12** | 19.15±1.92** |
| Metformin | 10.20±2.23**^##^ | 15.32±1.60**^##※※^ | 13.77±1.77*^##※^ | 12.33±1.51**^##^ | 10.78±1.59**^##^ |
| IOPH | 11.73±2.53**^##^ | 16.88±2.17**^##※※^ | 15.47±2.46**^##※^ | 14.37±2.25**^##^ | 12.78±2.18**^##^ |
| IOPM | 16.33±1.51**^▲▲^ | 19.10±1.87**#^▲▲※^ | 17.53±2.17**^#▲^ | 16.28±2.27**^#▲▲^ | 14.55±2.73**^#▲^ |
| IOPL | 16.67±1.27**^▲▲^ | 20.45±0.79**^#▲▲※※^ | 19.15±0.66**^#▲▲※※^ | 18.00±0.50**^#▲▲^ | 16.38±1.21**^#▲▲^ |

*VS* control group, * means significant difference (*p*<0.05); ** means extremely significant difference (*p*<0.01);

*VS* model group, # means significant difference; ## means extremely significant difference;

*VS* metformin group, ▲ means significant difference; ▲▲ means extremely significant difference;

*VS* 0 min，※means significant difference; ※※ means extremely significant difference;
